# Effector Mimics and Integrated Decoys, the Never-Ending Arms Race between Rice and *Xanthomonas oryzae*

**DOI:** 10.3389/fpls.2017.00431

**Published:** 2017-03-28

**Authors:** Paola Zuluaga, Boris Szurek, Ralf Koebnik, Thomas Kroj, Jean-Benoit Morel

**Affiliations:** ^1^INRA, UMR BGPI INRA/CIRAD/SupAgro, Campus International de Baillarguet,Montpellier, France; ^2^UMR – Interactions Plantes-Microorganismes-Environnement, IRD–Cirad–Université Montpellier, Institut de Recherche pour le Développement,Montpellier, France

**Keywords:** decoys, MIMICS, rice, *Xanthomonas*, Xa1, BED domain, Xo1, effector

## Abstract

Plants are constantly challenged by a wide range of pathogens and have therefore evolved an array of mechanisms to defend against them. In response to these defense systems, pathogens have evolved strategies to avoid recognition and suppress plant defenses (Brown and Tellier, 2011). Three recent reports dealing with the resistance of rice to *Xanthomonas oryzae* have added a new twist to our understanding of this fascinating co-evolutionary arms race (Ji et al., 2016; Read et al., 2016; Triplett et al., 2016). They show that pathogens also develop sophisticated effector mimics to trick recognition.

Transcription activator-like effectors (TALEs) are major virulence factors of *Xanthomonas* plant-pathogenic bacteria that modulate host transcription by *trans*-activating host susceptibility genes (Boch and Bonas, 2010). For this, TALEs possess a type III secretion signal mediating host cell translocation by the bacterial type III secretion system, nuclear localization signals (NLS) directing them into the plant nucleus and an activation domain that activates gene transcription in eukaryotic cells. In addition, TALEs possess a central repeat domain that mediates sequence specific DNA-binding. It is composed of variable numbers of a highly conserved 33–35 amino acid sequence in which residues 12 and 13, the so-called repeat variable di-residues (RVD), are hypervariable and determine the nucleotide binding specificity (Boch et al., 2009; Moscou and Bogdanove, 2009). Due to the discovery of the nucleotide binding specificity, a number of susceptibility genes targeted by *Xanthomonas* TALEs have been identified (Hutin et al., 2015a). Over the course of evolution, plants have acquired mutations in the promoter regions of susceptibility genes which abolish the binding and *trans*-activation by TALEs, hence leading to resistance by loss-of-susceptibility that is inherited in a recessive manner (Hutin et al., 2015b). Additionally, plants have evolved executor resistance genes whose transcription is induced by specific TALEs (upon binding to their promoters), resulting in a hypersensitive response, turning TALEs into avirulence (Avr) determinants (Zhang et al., 2015). The important role of the transcription machinery for TALE action in susceptibility and resistance is further highlighted by the recessive *xa5* resistance gene, a natural allele of the gene for the transcription factor IIA gamma subunit 5 (TFIIA γ5). Direct interaction of TALEs with TFIIAγ5 from rice activates disease susceptibility genes (Yuan et al., 2016).

These TALE-based dominant or recessive resistances to xanthomonads differ profoundly from standard pathogen resistance in plants that relies on the recognition of patterns or effectors by immune receptors. The best studied case of pattern recognition is the rice Xa21 receptor kinase that recognizes by direct binding RaxX, a sulfated peptide widely present in *Xanthomonas* that can therefore be considered a microbial pattern (Pruitt et al., 2015). Other examples are the NLRs (Nucleotide-binding domain, leucine-rich repeat) Xa1, Bs2, and Bs4 that, respectively, confer resistance to specific *Xanthomonas oryzae* pv. *oryzae* (*Xoo*) and *X. axonopodis* pv. *vesicatoria* isolates (Yoshimura et al., 1998; Tai et al., 1999; Schornack et al., 2004). NLRs are immune receptors commonly found in plants that display a canonical multi-domain structure. At the N-terminal they have coiled-coil or TIR (Toll-Interleukin Receptor) domains, a central nucleotide-binding domain and a C-terminal leucine-rich repeat domain and which recognize cytoplasmic effectors in a direct or indirect manner (Ellis, 2016).

A recent report (Ji et al., 2016) demonstrates that several TALEs are recognized by rice Xa1 protein, a member of the NLR family. Remarkably, two other groups reported the recognition of several TALE effectors by the Xo1 locus a yet uncharacterized gene, which the authors argue to be likely a NLR protein (Read et al., 2016; Triplett et al., 2016). This rather non-specific recognition of TALEs does not lead to broad-spectrum resistance to *Xoo* and *X. oryzae* pv. *oryzicola* (*Xoc*) because TALE-derived (truncTALES and iTALES) effectors can suppress this resistance suggesting they might act as effector mimics. In the case of Xo1 this suppression is independent from DNA binding at least for the Tal2h truncTALE (Read et al., 2016). It will be interesting to determine whether Xa1 inhibition by iTALES is independent of DNA binding as well, or if its suppression is by a different mechanism. These findings provide an exciting novel insight into the evolutionary arms race between plants and pathogens and reveals new functions of TALEs. In these studies, the function of different combinations of TALEs was evaluated by reintroducing them into *Xoo* strains depleted for most TALEs (Ji et al., 2016; Triplett et al., 2016). The first discovery was that both *Xa1* and the newly identified *Xo1* locus trigger resistance by recognizing several unrelated *X. oryzae* TALEs that differ in their target sequence and their number of central repeats (Ji et al., 2016; Triplett et al., 2016). The structural motifs that are recognized and the mode of recognition, either direct or indirect, are not yet defined. However, it appears that at least 3.5 central repeats regardless of their RVDs are required to trigger both Xa1 and Xo1 resistance (Ji et al., 2016; Triplett et al., 2016). Additionally, in the case of Xo1, TALE recognition and activation of resistance does not require the activation domain and is independent of DNA-binding (Read et al., 2016; Triplett et al., 2016). As a consequence, Xa1 recognizes an entire effector family, and not as other NLRs, only individual effectors in a very specific manner. This is a new feature of NLR activity that has previously been rather associated with membrane bound receptor complexes which can in certain cases recognize entire, widely distributed effector families (Böhm et al., 2014).

The second major discovery of these studies is that truncated TALE gene variants, previously considered as pseudogenes, designated as truncTALEs (Read et al., 2016) can act as interfering TALEs (iTALEs; Ji et al., 2016). Thus, some truncTALEs can block *Xa1*- and *Xo1*-mediated recognition of full-length TALEs, hence acting as iTALEs and suppressing resistance. These iTALEs/truncTALEs are characterized by specific deletions in the conserved N- and C-terminal sequences, require at least 3.5 central repeats and do not depend on specific RVDs, suggesting that their activity does not rely on DNA-binding or the direct regulation of the transcription of target genes. As suggested by Read et al. (2016) at least in the case of Xa1 it is tempting to speculate that iTALEs compete with full-length TALEs for binding to the NLR receptor but, on the contrary of the genuine ligand, do not activate the immune receptor, thus acting as dominant suppressors. Suppressors of NLR-mediated resistance have been identified in various phytopathogenic organisms but in the cases where they have been molecularly identified they correspond to effectors that are unrelated to the recognized Avr effectors (Houterman et al., 2008; Bourras et al., 2015; Plissonneau et al., 2016). A completely new and extraordinary twist in microbial virulence comes from the discovery that the oomycete pathogen *Phytophthora sojae* deploys an effector mimic PsXLP1 which resembles the functional virulence protein PsXEG1 to disrupt plant defense (Ma et al., 2017). The discovery of iTALEs/truncTALEs suggests that *Xoo* and *Xoc* also deploy effectors that mimic other, recognized effectors. Similarly to PsXLP1/PsXEG1, iTALEs/truncTALEs can be viewed as effector mimics that the pathogen uses to interfere with recognition by the plant. These two examples open novel exciting dimensions in the understanding of plant–pathogen co-evolution. Whether other effectors act in a similar way, as suppressors of resistance, is an unexplored question and may force us to consider the large effector repertoires with a completely new perspective in which some effectors may in fact be effector mimics.

How TALEs are recognized by Xa1 and Xo1, directly or indirectly, and how truncTALEs/iTALEs interfere with recognition is unknown. However, for the case of Xa1, it is tempting to speculate that it might involve the BED-type zinc finger domain which is integrated in the N-terminal region of this NLR protein (Kroj et al., 2016). In fact, we and others showed recently that unconventional integrated domains in NLRs are involved in the detection of effectors either by direct binding or by posttranslational modifications; thus these integrated domains may mimic the true effector target proteins and therefore act as integrated decoys (Cesari et al., 2013, 2014; Le Roux et al., 2015; Maqbool et al., 2015; Sarris et al., 2015, 2016; Kroj et al., 2016). The BED domain has been shown to bind DNA and is present in transposases and transcription factors (Hayward et al., 2013). ZBED, a rice protein containing three BED domains was recently shown to be required for full resistance to the rice blast fungus (Kroj et al., 2016), suggesting a role of BED proteins in plant–pathogen interactions. It could therefore be that TALEs recruit BED proteins as co-factors for the *trans*-activation of target genes and are trapped by Xa1 according to the integrated decoy model (e.g., by binding directly the BED domain of Xa1). iTALEs may interfere with TALE recognition in a dominant-negative manner by occupying some binding sites of Xa1 for TALEs and by this outcompete TALEs for Xa1-binding without triggering receptor activation (**Figure 1**). This model is consistent with the finding of Ji et al. (2016) that Xa1-mediated TALE recognition and iTALE-mediated suppression of resistance require nuclear localization of TALEs and iTALEs. By contrast, Read et al. (2016) report that deleting the putative NLS in the iTALE Tal2h does not affect its ability to suppress Xo1. Yet, the localization of the Tal2h NLS mutant was not analyzed and we cannot rule out that Xo1-mediated TALE recognition and truncTALE suppression differs from Xa1 and can happen in the cytoplasm or in the nucleus in a NLS-independent manner. It will therefore be particularly interesting to investigate the location of Xa1 and Xo1, and to determine whether Xo1 is a NLR protein to gain a better insight into the potential similarities or differences of both.

**FIGURE 1:**
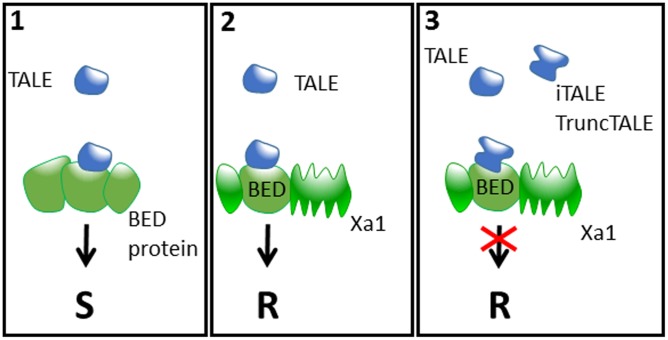
**A speculative model for the arms race between plants and *Xanthomonas.*** (1) *Xanthomonas* bacteria secrete TALEs into plant cells that presumably target protein(s) containing BED domain(s) to enhance susceptibility (S). (2) Plants have evolved immune receptors such as Xa1 that contain a BED domain which behaves as an integrated decoy, thus leading to TALE recognition and triggering resistance (R). (3) In turn, *Xanthomonas* evolved iTALEs and TruncTALEs, which are derivatives of full-length TALEs recognized by Xa1. These iTALEs and TruncTALEs interact directly or indirectly with Xa1 to inhibit its function, thus re-establishing susceptibility even in the presence of full-length TALEs.

The integrated decoy model for NLRs is a valuable concept to generate several hypotheses that can be challenged experimentally but that are entirely speculative at this point: Is the BED domain of Xa1 required for TALE recognition and iTALE-mediated suppression and if so, do these activities rely on direct binding between the BED domain and certain motifs in the TALE? Do TALEs interact with other Xa1 domains? Do TALEs interact directly or indirectly with other BED domain proteins and do such interactions contribute to target gene *trans*-activation? If yes, it is expected that mutants of these BED domain proteins are less susceptible to xanthomonads that rely on TALEs for virulence. Finally, a better molecular (structural) understanding of how TALEs and iTALEs interact with Xa1 may allow engineering NLR receptors that recognize full-length TALEs but not iTALEs and thus would truly confer broad-spectrum resistance against *Xoo* and *Xoc*.

## Author Contributions

PZ, BS, RK, TK, and J-BM participated to the writing of this mini-review.

## Conflict of Interest Statement

The authors declare that the research was conducted in the absence of any commercial or financial relationships that could be construed as a potential conflict of interest.

## References

[B1] Boch J. Bonas U. (2010). Xanthomonas AvrBs3 family-type III effectors: discovery and function. *Annu. Rev. Phytopathol.* 48 419–436. 10.1146/annurev-phyto-080508-081936 19400638

[B2] Boch J. Scholze H. Schornack S. Landgraf A. Hahn S. Kay S. (2009). Breaking the code of DNA binding specificity of TAL-type III effectors. *Science* 326 1509–1512. 10.1126/science.1178811 19933107

[B3] Böhm H. Albert I. Oome S. Raaymakers T. M. Van den Ackerveken G. Nürnberger T. (2014). A conserved peptide pattern from a widespread microbial virulence factor triggers pattern-induced immunity in Arabidopsis. *PLoS Pathog.* 10:e1004491 10.1371/journal.ppat.1004491 PMC4223075 25375108

[B4] Bourras S. McNally K. E. Ben-David R. Parlange F. Roffler S. Praz C. R. (2015). Multiple avirulence loci and allele-specific effector recognition control the *Pm3* race-specific resistance of wheat to powdery wildew. *Plant Cell* 27 2991–3012. 10.1105/tpc.15.00171 26452600 PMC4682313

[B5] Brown J. K. Tellier A. (2011). Plant-parasite coevolution: bridging the gap between genetics, and ecology. *Annu. Rev. Phytopathol.* 49 345–367. 10.1146/annurev-phyto-072910-095301 21513455

[B6] Cesari S. Bernoux M. Moncuquet P. Kroj T. Dodds P. N. (2014). A novel conserved mechanism for plant NLR protein pairs: the “integrated decoy” hypothesis. *Front. Plant Sci.* 5:606 10.3389/fpls.2014.00606 PMC4246468 25506347

[B7] Cesari S. Thilliez G. Ribot C. Chalvon V. Michel C. Jauneau A. (2013). The rice resistance protein pair RGA4/RGA5 recognizes the *Magnaporthe oryzae* effectors AVR-Pia and AVR1-CO39 by direct binding. *Plant Cell* 25 1463–1481. 10.1105/tpc.112.107201 23548743 PMC3663280

[B8] Ellis J. G. (2016). Integrated decoys and effector traps: how to catch a plant pathogen. *BMC Biol.* 14:13 10.1186/s12915-016-0235-8 PMC4759852 26896088

[B9] Hayward A. Ghazal A. Andersson G. Andersson L. Jern P. (2013). ZBED evolution: repeated utilization of DNA transposons as regulators of diverse host functions. *PLoS ONE* 8:e59940 10.1371/journal.pone.0059940 PMC3606216 23533661

[B10] Houterman P. M. Cornelissen B. J. Rep M. (2008). Suppression of plant resistance gene-based immunity by a fungal effector. *PLoS Pathog.* 4:e1000061 10.1371/journal.ppat.1000061 PMC2330162 18464895

[B11] Hutin M. Perez-Quintero A. L. Lopez C. Szurek B. (2015a). MorTAL Kombat: the story of defense against TAL effectors through loss-of-susceptibility. *Front. Plant Sci.* 6:535 10.3389/fpls.2015.00535 PMC4500901 26236326

[B12] Hutin M. Sabot F. Ghesquière A. Koebnik R. Szurek B. (2015b). A knowledge-based molecular screen uncovers a broad-spectrum OsSWEET14 resistance allele to bacterial blight from wild rice. *Plant J.* 84 694–703. 10.1111/tpj.13042 26426417

[B13] Ji Z. Ji C. Liu B. Zou L. Chen G. Yang B. (2016). Interfering TAL effectors of *Xanthomonas oryzae* neutralize *R*-gene-mediated plant disease resistance. *Nat. Commun.* 7:13435 10.1038/ncomms13435 PMC5097170 27811915

[B14] Kroj T. Chanclud E. Michel-Romiti C. Grand X. Morel J. B. (2016). Integration of decoy domains derived from protein targets of pathogen effectors into plant immune receptors is widespread. *New Phytol.* 210 618–626. 10.1111/nph.13869 26848538 PMC5067614

[B15] Le Roux C. Huet G. Jauneau A. Camborde L. Tremousaygue D. Kraut A. (2015). A receptor pair with an integrated decoy converts pathogen disabling of transcription factors to immunity. *Cell* 161 1074–1088. 10.1016/j.cell.2015.04.025 26000483

[B16] Ma Z. Zhu L. Song T. Wang Y. Zhang Q. Xia Y. (2017). A paralogous decoy protects Phytophthora sojae apoplastic effector PsXEG1 from a host inhibitor. *Science* 17 355 710–714. 10.1126/science.aai7919 28082413

[B17] Maqbool A. Saitoh H. Franceschetti M. Stevenson C. E. M. Uemura A. Kanzaki H. (2015). Structural basis of pathogen recognition by an integrated HMA domain in a plant NLR immune receptor. *eLife* 4:e08709 10.7554/eLife.08709.001 PMC4547098 26304198

[B18] Moscou M. J. Bogdanove A. J. (2009). A simple cipher governs DNA recognition by TAL effectors. *Science* 326 1501 10.1126/science.1178817 19933106

[B19] Plissonneau C. Daverdin G. Ollivier B. Blaise F. Degrave A. Fudal I. (2016). A game of hide and seek between avirulence genes *AvrLm4-7* and *AvrLm3* in *Leptosphaeria maculans*. *New Phytol.* 209 1613–1624. 10.1111/nph.13736 26592855

[B20] Pruitt R. N. Schwessinger B. Joe J. Thomas N. Liu F. Albert M. (2015). The rice immune receptor XA21 recognizes a tyrosine-sulfated protein from a Gram-negative bacterium. *Sci. Adv.* 1:e1500245 10.1126/sciadv.1500245 PMC4646787 26601222

[B21] Read A. C. Rinaldi F. C. Hutin M. He Y. Q. Triplett L. R. Bogdanove A. J. (2016). Suppression of *Xo1*-mediated disease resistance in rice by a truncated, non-DNA-binding TAL effector of *Xanthomonas oryzae*. *Front. Plant Sci.* 7:1516 10.3389/fpls.2016.01516 PMC5062187 27790231

[B22] Sarris P. F. Cevik V. Dagdas G. Jones J. G. D. Krasileva K. V. (2016). Comparative analysis of plant immune receptor architectures uncovers host proteins likely targeted by pathogens. *BMC Biol.* 14:8 10.1186/s12915-016-0228-7 PMC4759884 26891798

[B23] Sarris P. F. Duxbury Z. Huh S. U. Ma Y. Segonzac C. Sklenar J. (2015). A plant immune receptor detects pathogen effectors that target WRKY transcription factors. *Cell* 161 1089–1100. 10.1016/j.cell.2015.04.024 26000484

[B24] Schornack S. Ballvora A. Gürlebeck D. Peart J. Ganal M. Baker B. (2004). The tomato resistance protein Bs4 is a predicted non-nuclear TIR-NB-LRR protein that mediates defense responses to severely truncated derivatives of AvrBs4 and overexpressed AvrBs3. *Plant J.* 37 46–60. 10.1046/j.1365-313X.2003.01937.x 14675431

[B25] Tai T. H. Dahlbeck D. Clark E. T. Gajiwala P. Pasion R. Whalen M. C. (1999). Expression of the Bs2 pepper gene confers resistance to bacterial spot disease in tomato. *Proc. Natl. Acad. Sci. U.S.A.* 96 14153–14158. 10.1073/pnas.96.24.14153 10570214 PMC24206

[B26] Triplett L. R. Cohen S. P. Heffelfinger C. Schmidt C. L. Huerta A. I. Tekete C. (2016). A resistance locus in the American heirloom rice variety Carolina Gold Select is triggered by TAL effectors with diverse predicted targets and is effective against African strains of *Xanthomonas oryzae* pv. *oryzicola.* *Plant J.* 87 472–483. 10.1111/tpj.13212 27197779 PMC5030141

[B27] Yoshimura S. Yamanouchi U. Katayose Y. Toki S. Wang Z. X. Kono I. (1998). Expression of *Xa1*, a bacterial blight-resistance gene in rice, is induced by bacterial inoculation. *Proc. Natl. Acad. Sci. U.S.A.* 95 1663–1668. 10.1073/pnas.95.4.1663 9465073 PMC19140

[B28] Yuan M. Ke Y. Huang R. Ma L. Yang Z. Chu Z. (2016). A host basal transcription factor is a key component for infection of rice by TALE-carrying bacteria. *eLife* 5:e19605 10.7554/eLife.19605 PMC4993585 27472897

[B29] Zhang J. Yin Z. White F. (2015). TAL effectors and the executor *R* genes. *Front. Plant Sci.* 6:641 10.3389/fpls.2015.00641 PMC4542534 26347759

